# Correspondence on “Endocytosis-Mediated Transport of Pb in Rat Blood Cells”

**DOI:** 10.1021/acs.est.3c06212

**Published:** 2023-09-28

**Authors:** Ingvar A. Bergdahl

**Affiliations:** Umeå University, Department of Public Health and Clinical Medicine, Division of Sustainable Health, 90187 Umeå, Sweden

It was with great interest that I read the article ‘Endocytosis-Mediated Transport of Pb in Rat Blood Cells’ by Guo et al.^1^ The authors aimed to look for molecular targets for lead transport by exploring lead-binding proteins in rat blood, and they hoped that this can provide a possible strategy for reducing blood-lead levels in normal human beings. I comment on the studies of blood cells, as knowledge about old results from humans, as well as about some technical and biochemical issues, appears to be unknown to the authors and may affect the interpretation of the observations reported.

Guo et al.^1^ used size-exclusion chromatography (SEC) to separate proteins, coupled to inductively coupled plasma mass spectrometry (ICP-MS) for detection of lead. Only <1% of the lead in blood cells then eluted bound to macromolecules, while >99% eluted with the ion front. After further purification by sodium dodecyl sulfate–polyacrylamide gel electrophoresis (SDS–PAGE), the authors identified a number of proteins that had eluted together with lead.

SEC-ICP-MS has previously been used for human erythrocytes, showing that the main lead-binding protein, as suggested by Sakai et al.,^2^ is δ-aminolevulinic acid dehydratase (ALAD), a 250 kDa homo-octameric zinc-binding enzyme known to be inhibited by lead. ALAD accounted for ∼80% of erythrocyte lead at moderate lead levels^3^ but considerably less at high lead concentrations.^4^ No lead was observed to elute with the ion front. The identity of the protein was confirmed by use of ALAD antibodies.^5^ Reviews of this can be found in major lead overviews^6,7^ and elsewhere.^8−10^ Before 1998, it was common to believe that lead was mainly bound to hemoglobin, but that was a misconception. In contrast, Guo et al. observed no binding of lead to ALAD (or hemoglobin) in rats.^1^

A major issue in studies of metal-binding proteins is preserving the native metal–protein associations.^11^ Lead ions are coordinated to proteins. They are not covalently bound but bound as ligands in positions where the metal ion is held by amino acids with negative polarity at suitable angles and distances.^12,13^ A protein’s lead-binding properties therefore depend on its tertiary structure. This tertiary structure can be altered by both sample preparation and interaction with the gel surface or buffer when passing through a chromatographic column. Loss of lead from proteins to the gel and mobile phase, as well as uptake of lead by proteins, appears to occur.^3^ It is therefore advisable to keep track of the lead recovery through the process, for example, by placing a flow injection valve between the separation column and the ICP-MS instrument, as described by Owen et al.,^14^ and using that for injection of a calibration standard. In addition to providing quantitation of lead peaks and evaluation of the recovery from the column, the repeated calibration allows the loss of sensitivity caused by salt build-up on the ICP-MS cones to be monitored. To minimize such a build-up, sodium salts are often avoided in the mobile phase of a SEC-ICP-MS system. We used ammonium hydrogen carbonate, Bishop et al.^11^ recommended ammonium acetate buffers. Guo et al.^1^ instead used sodium salts and a high-matrix introduction mode for salt-rich matrices, as described by Tang et al.^15^ Whether that method for salt-rich matrices provides any advantages when ammonium buffers are used appears not to have been tested yet.

With regard to identification of a lead-binding protein, we used ALAD-specific antibodies to extract the protein from the sample and could then observe the disappearance of the largest lead peak.^5^ The method applied for protein identification by Guo et al.^1^ is not as conclusive. The authors took the protein samples that were identified by ICP-MS as carrying lead and subjected them to SDS–PAGE, and the protein bands corresponding to the expected molecular weight were cut and sent for mass spectrometry analysis. This is not an advisable method because (i) native lead binding is not expected to be preserved in SDS, due to the denaturation of the protein, so it is uncertain if the proteins within the band that is cut out truly bind to lead *in vivo* and (ii) molecular masses can change (for example, ALAD will appear ∼250 kDa in SEC, but as it is an octamer, it will appear at ∼30 kDa on SDS–PAGE after denaturation).

From the information presented above, it appears that Guo et al. used SEC conditions that preserved the native metal–protein association for <1% of the lead and applied a not very conclusive method to identify proteins co-eluting with that <1% of the lead. The other >99% of the lead was left unnoticed. This is a weak foundation for a hypothesis, but on the basis of functions found for these proteins, they tested if injection of Dynasore, an endocytosis inhibitor, into rats decreases the rate of uptake of lead from the gut into blood. It did. Whether that effect is related to lead binding of proteins in blood cells must be regarded as uncertain. In addition, while uptake of lead from the gut is unwanted, it should be noticed that transport of lead into cells is not necessarily bad. Lead binding to intracellular proteins may provide protection of organs against toxicity, which has been discussed by Gonick.^9^

Surprisingly little is known about the mechanisms of lead toxicity (for a discussion, see ref (10) and references therein). Studies of lead-binding proteins can be a way to learn more about the transport and toxicity of lead. I hope that this comment can prevent overinterpretation of the observations presented by Guo et al.^1^ and that the references provided herein can inspire and guide authors and readers of the article to take advantage of previously published insights and technical “tips and tricks” and expand the knowledge in the fascinating, difficult, and underexplored area of lead-binding proteins. A combination with modern biochemical methods is strongly desired, and I appreciate and recognize Guo et al. for going in that direction.
